# Assessment of Sun Protection Knowledge and Behaviors of US Youth

**DOI:** 10.1001/jamanetworkopen.2021.34550

**Published:** 2021-11-15

**Authors:** Arianna Strome, Kelsey Herbert, Kevin Walsh, Olivia Lamberg, Marika E. Waselewski, Tammy Chang

**Affiliations:** 1 University of Michigan Medical School, Ann Arbor; 2 Department of Family Medicine, University of Michigan, Ann Arbor; 3 Institute of Healthcare Policy and Innovation, University of Michigan, Ann Arbor

## Abstract

This survey study examines sun protection knowledge and behaviors among US youth aged 14 to 24 years.

## Introduction

Skin cancer is the most common cancer in the United States, and the incidence continues to rise. Greater than 5 lifetime sunburns double one’s risk of melanoma.^1^ Skin protection among youth is the most effective means to prevent new cases of melanoma and nonmelanoma skin cancers. Despite this, less than one-third of youth aged 11 to 18 years routinely use sun protection.^2^

Efforts are underway to increase the priority of sun protection among youth.^3,4^ Changing sun exposure behaviors is difficult to implement^4^ and even those who do use sunscreen often do so incorrectly.^5^ The goal of this study is to identify youths’ knowledge and experiences using sun protection and to understand what interventions would be most beneficial in increasing the use of sun protection among their peers.

## Methods

This survey study used MyVoice, a national text message–based polling platform of youth. The University of Michigan institutional review board approved this study with a waiver of parental consent for minor participants as this study was deemed of minimal risk to participants. This study followed the American Association for Public Opinion Research (AAPOR) reporting guideline for survey research. MyVoice participants ranged from age 14 to 24 years and were recruited from social media based on national benchmarks from the American Community Survey. Race and ethnicity data were self-reported. Participants were given $1 for responding to each week’s survey topic.^6^

Five open-ended probes focused on sun protection were sent to 1151 youths on April 9, 2021. Two investigators created a codebook based on salient topics and independently analyzed each question. Discrepancies in coding were resolved by a third investigator. Prevalence of themes was summarized using descriptive statistics. Statistical analyses were performed using SAS version 9.4 (SAS Institute) on May 23, 2021.

## Results

Our survey had an 84.9% response rate (977 of 1151). Among 977 respondents included in this study, 473 (48.1%) identified as female, 104 (10.7%) as Hispanic, 64 (6.6%) as non-Hispanic Black, and 607 (62.2%) as non-Hispanic White; the mean (SD) age was 19.3 (2.4) years (Table 1).

**Table 1.** Respondent Demographic Characteristics

Characteristic	Respondents, No. (%) (N = 977)
Age, y	
Mean (SD)	19.3 (2.4)
14-17	241 (24.7)
18-24	736 (75.3)
Gender identity	
Female	473 (48.1)
Male	403 (41.3)
Other^a^	101 (10.3)
Race and ethnicity	
Hispanic	104 (10.7)
Non-Hispanic	
Black	64 (6.6)
White	607 (62.2)
Other^b^	201 (20.6)
Education	
High school or less	398 (40.7)
Some college or tech school	403 (41.2)
College or tech graduate	176 (18.0)
Region	
Midwest	317 (32.8)
Northeast	178 (18.4)
South	264 (27.3)
West	207 (21.4)
Received free or reduced lunch	
Yes	374 (38.5)
No	598 (61.5)

^a^ Other gender included those who self-reported identifying as transgender (FTM), transgender (MTF), nonbinary, and other (please specify).

^b^ Races included in the non-Hispanic other category include those who self-reported the following races: American Indian or Alaska Native; Asian; Native Hawaiian or other Pacific Islander; other (please describe).

When asked how important it is to protect their skin from the sun, 62.1% (594 of 957) stated very important, 25.5% (244 of 957) stated important, and 5.4% (54 of 957) stated somewhat important. Skin cancer was the most frequently cited reason for sun protection (51.7%; 495 of 957). Although 90.1% of youth (840 of 932) reported use of sunscreen, 81.1% (751 of 926) noted having had 1 or more sunburns, with 28.4% (263 of 926) reporting 5 or more. To increase the use of sun protection among youth, respondents suggested demonstrating the consequences of sun exposure (41.1%; 405 of 932), using traditional media (16.8%; 165 of 932), and increasing product accessibility (10.6%; 104 of 932) (Table 2).

**Table 2.** Representative Quotes

Question, theme	Respondents, No. (%)	Example participant quote
Question 1a: How important do you think it is to protect your skin from the sun? (n = 957)		
Very important	594 (62.1)	“It’s extremely important to prevent skin aging and cancer”
Important	244 (25.5)	“It’s quite important but not the utmost top priority. I know it’s important to protect yourself from skin cancer and ageing effects.”
Somewhat important	52 (5.4)	“Only important if you’re going to be exposed for a long time to heavy sun”
Unimportant	50 (5.2)	“Not really important because it is the bottom priority of danger”
Question 1b: Why?		
Skin cancer	495 (51.7)	“It’s pretty important because the UV rays can cause skin cancer”
Health/disease	172 (18.0)	“It’s very important to [protect your skin from the sun]. Later in life, you can end up with badly damaged skin or even skin cancer in extreme cases.”
Sunburn	144 (15.0)	“To keep from sunburn”
Photoaging	114 (11.9)	“Very important because damage builds and when you are old your skin will show it”
		“It’s really important. To prevent skin cancer but also aging.”
Question 2: When do you protect yourself from the sun? (n = 902)		
Extended time outside	298 (33.0)	“When I know I’ll be outside for an extended period of time”
Sunshine	229 (25.4)	“When I’m exposed to heavy sun”
Water	209 (23.2)	“When I go out to the beach/outside in the sun for a while”
Summer	140 (15.5)	“In the summer if I am going to be outside a lot”
Any time outside	135 (15.0)	“Whenever I go outside”
Every day	121 (13.4)	“I wear spf on my face every day…”
Question 3: How do you protect yourself from the sun? (n = 932)		
Sunscreen	840 (90.1)	“Sunscreen”
Hat	208 (22.3)	“Wear a hat”
Clothing	201 (21.6)	“Wear long clothing”
Shade	118 (12.7)	“Stand in the shade”
Sunglasses	101 (10.8)	“Sunglasses”
Outdoor avoidance	64 (6.9)	“Try not to be outside for long periods of time when the UV index is really high”
Moisturizer	47 (5.0)	“I use a daily moisturizer with spf on my face”
Nothing	25 (2.7)	“Literally nothing”
Question 4a: How many red, painful, or peeling sunburns have you had in your life? (n = 926)		
None	172 (18.6)	“none”
Few (1-4)	488 (52.7)	“a few”
Many (≥5)	263 (28.4)	“Many, I burn easily”
Question 4b: If so, have they ever blistered? (n = 486)		
Blistered	189 (38.9)	“Maybe 2-3 have blistered”
Not blistered	297 (61.1)	“They have never blistered”
Question 5: What could be done to encourage more people to use sun protection? (n = 895)		
Consequences	405 (41.1)	“Showing people the long term effects”
Traditional media	165 (16.8)	“Perhaps advertising the importance of it more often”
Product accessibility	104 (10.6)	“Free Sunscreen for those who can’t afford”
Product attractiveness	84 (8.5)	“Making sunscreen smell better”
Education	75 (7.6)	“Better teaching about skin diseases in primary education”
Unsure	83 (8.4)	“I have no clue I don’t have that problem”

## Discussion

Our findings suggest that youth understand the short and long-term risks of sun damage yet have difficulty successfully implementing sun protection. Despite nearly all youth (90.1%) stating they use sunscreen, the high number of self-reported burns suggests public health personnel and clinicians must change their approach.

This study had some limitations. Although text messaging allows us to elicit the open-ended responses of youth, it does not allow us to engage in a 2-way dialogue to clarify any responses. Also, although our sample is nationwide, it is not nationally representative, which may limit generalizability.

This study adds to current literature by revealing what may be preventing US youth from using consistent sun protection. Based on youths’ desire for increased product accessibility, cost and inconvenience are likely barriers preventing consistent sunscreen use. Approximately 40% believe poor sun protection behaviors would improve with education specifically illustrating the consequences of sun damage. Strategies suggested by youth to increase the use of sun protection include increasing sunscreen accessibility, widely distributed media campaigns, and improved government policies to strengthen sun protection standards and education in schools and workplaces. Implementing these strategies suggested by youth may help prevent sunburns during childhood and adolescence and, ultimately, decrease the risk of skin cancer later in life.
